# Simple Design of Broadband Polarizers Using Transmissive Metasurfaces for Dual Band Ku/Ka Band Applications

**DOI:** 10.3390/s22239152

**Published:** 2022-11-25

**Authors:** Ayesha Kosar Fahad, Rabia Nazir, Cunjun Ruan

**Affiliations:** 1 School of Electronics & Information Engineering, Beihang University, Beijing 100191, China; 2 Electrical Engineering Department, University of Engineering & Technology (UET), Lahore 54890, Pakistan; 3 Beijing Key Laboratory for Microwave Sensing & Security Applications, Beihang University, Beijing 10091, China

**Keywords:** dual band, linear-to-circular converter, periodic array

## Abstract

Major challenges affecting polarizers for communication systems include the inability to perform over a wide bandwidth with a simple design. Orthogonal outgoing polarization for polarization-diverse applications and stable performances for oblique incidence angles are also major requirements. This paper presents the design of a polarizer that can perform over a wide range of bandwidths in dual frequency bands. The unit cell is uniquely designed using a split circular ring resonator enclosed in a square ring with the addition of three-square patches. As a result, the incoming linearly polarized x(y) wave is converted into a transmitted LHCP (RHCP) wave in the Ku and Ka bands. The operational bandwidths are 11.05~16.75 GHz (41%) and 34.16~43.03 GHz (23%). The proposed polarizer is ultrathin, works in dual wide-bands, is polarization-diverse, and maintains performance over ±45° and ±30° oblique incidences, which makes it a strong candidate for many communication systems.

## 1. Introduction

Wireless sensor networks (WSNs) are used in wireless communication systems for various applications, such as smart cities, wireless monitoring, military surveillance, and medical imaging [1,2,3]. The polarization of an electromagnetic wave represents the direction of an oscillating electric field while traveling through any channel. Manipulation and control of the polarization state have many applications in wireless communication systems, such as satellite communication, remote sensing, and stealth technology [4]. In communication systems, the choice of antenna depends upon the applications and medium. In these systems, problems due to Faraday rotation, polarization mismatch, and multipath fading result in the degradation of the performance of channel. Therefore, circular polarized (CP) waves are used in these cases instead of linearly polarized (LP) waves; in particular, in communication systems and global navigation systems (GNSs). Obtaining CP waves in dual-wide-band for multiband operation is a challenging task.

There are essentially two methods to obtain CP waves. The first is by employing CP antennas using the direct generation phenomenon. Dipoles and spiral and helical antennas are examples of such antennas [5,6,7]. The other method of obtaining CP waves is using a transformation method for the polarization state: so-called polarization conversion. CP antennas based on the generation phenomenon contain large and bulky elements, which are usually cascaded with built-in polarizers [8], making these antennas complex structures. Furthermore, CP antennas, once fabricated, cannot be used as other types of antennas. In contrast, obtaining CP waves using the polarization conversion method allows antennas to perform both as LP and CP antennas. If required, an LP antenna with linearly polarized characteristics alone can be used, and when CP wave characteristics are required, an LP antenna along with a polarization converter can be used. Furthermore, LP antennas are simpler to design.

Conventional polarization converters (polarizers) are bulky and often have narrow operational bandwidths. These converters usually consist of birefringence wave-plates and liquid crystals [9,10,11,12], which are quite complex. To simplify and miniaturize the polarizers, the concept of polarization conversion has been developed using frequency-selective surfaces [13,14,15], metamaterials [16,17], and metasurfaces [18,19]. These polarizers have also been developed based on a reflection mode, which causes feed blockages [20]. Therefore, polarizers operating in transmission mode are desired. Existing transmissive polarizers are based on a single wide-band of operations [21,22]. In satellite communication systems with non-adjacent transmit and receive channels lying at two distinct bands, dual-band polarization conversion can play an important role. Further, the need for an orthogonal polarization direction with high isolation between the transmit and receive signals requires an orthomode transducer (OMT), which makes the system bulky. A dual-band, dual-polarized converter may be an ideal candidate for such applications, removing the need for an OMT and making the system less complex. Furthermore, a dual-band polarization conversion operation is required for the merging of multiple systems to achieve adequate volume and size reductions. Thus, in the last couple of years, researchers have worked on dual-band polarizers [23,24,25,26,27,28,29,30,31] using multi-layered [24,29,31], bi-metallic layered [23,25,26,28,30], and single-layered structures [27].

Different periodic-structure unit cell elements have been used for dual-band polarizers. Wang et al. [28] proposed dual-band polarization converters using JC-based structures. The structures were bi-layered and dual-band performance with 24% and 11% bandwidths was achieved. Kaiyue et al. [31] reported a polarization converter in X and Ku bands using double split-ring layers with a central rectangular patch. They reported 6.4% and 2.1% bandwidths using a tri-layered structure. However, for a structure consisting of more than two metallic layers, the fabrication process becomes complex, requiring alignment to be perfectly matched. Furthermore, the operating band for polarization conversion is narrow and loses stability with variation in the incident waves’ angles. Therefore, wide operational bands, simple design and fabrication, and stability over a wide range of oblique incidences are key requirements for dual-band polarizers.

In this work, a dual-band polarizer converter was developed using a unique design strategy that can be used in communication systems to generate dual-band CP waves from linearly polarized antennas in wide frequency bands. The proposed structure has wide operational bands, is uni-layered and polarization-diverse, and remains stable over a wide range of oblique incidences of linearly polarized waves.

This communication is structured as follows. In Section 2, the design principle for the metasurface-based structure is described. The simulation and detailed physical analyses are described in Section 3. A discussion of the experimental results is carried out in Section 4, along with a comparison of the performance with other state-of-the-art designs. Finally, a conclusion is presented in Section 5.

## 2. Materials and Methods

**Figure 1:**
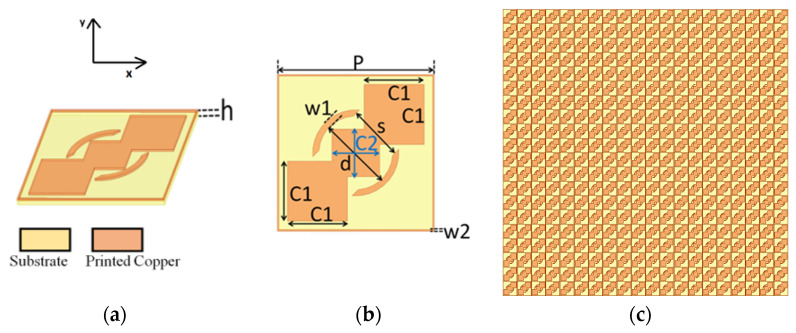
Unit cell for the proposed structure: (**a**) perspective 3D view; (**b**) top view; (**c**) 2D array.

In this work, we followed a unique design idea for transmission-based wide-band polarization-converting metasurfaces, described as follows:

1. Ghosh et al. used a square ring as a basic building block for a periodic structure with wide operational bandwidth without performing any polarization conversion operations [32]. Therefore, the first element in the unit cell was a square ring with a width *w*2 in order to obtain wider operational bandwidth, as shown in Figure 1. The width of the ring controls the bandwidth, as well as the separation between the two bands of operation;
2. Multiple square patches on a unit cell can cause multiple resonances, resulting in multiple bands of operation. Therefore, placing these patches along the diagonal can result in multiple operational bands [33]. In this way, three squares, *C*_1_, *C*_2_ and *C*_1_, were placed diagonally;
3. According to the concept from [25,26,27], a split circular ring along the diagonal split can enable polarization conversion operations. Therefore, a diagonal split ring with internal diameter *d* and split opening *S* was selected as the third element in the unit cell. The width of the ring was *w*1. The effect of each parameter on the design of the polarizer is described in the following paragraph.

Using the above-described design idea, the unit cell of the complete metasurfaces-based structure is as shown in Figure 1. It contains a square ring with metallic width *w*2. Three diagonal square patches were adopted for the dual-band polarization conversion operation. A circular ring with a diagonal split allows polarization conversion. In Figure 1, the periodicity of the proposed unit cell is represented with *P*. *w*1 represents the width of the circular ring with the split length *S* along the diagonal. The outer square patches have the sizes *C*1 *× C*1, whereas the inner square patch has the size *C*2 *× C*2. The split circular ring has the diameter *d*. The thickness of the substrate is represented as *h*. The parameters *C*1, *C*2, *d*, *S*, *w*1, *w*2, and *P* were optimized to obtain a polarization conversion operation from 11.05 to 16.75 GHz and 34.16 to 43.03 GHz. The parameter *P* directly controls the frequency of the operation. The dimensions of squares *C*1 and *C*2 control the separation of the two operating frequency bands. Parameters *d* and *S* control the polarization conversion due to the break in the isotropicity of the structure. The width of the square ring *w*2 controls the performance of the conversion and the bandwidth of the bands. The final optimized parameters of the proposed structure are tabulated in Table 1.

## 3. Simulation and Analysis

Using the described design idea, simulation for the proposed structure was carried out using the electromagnetic simulation tool Ansys Electronics. Floquet ports, along with master–slave boundary conditions, were applied to simulate the periodic structure.

Let us analyze the proposed structure for an incident horizontally polarized (x-polarized) wave. Since the unit cell is uni-diagonal symmetric (anisotropic structure), it transmits a cross-polarization component of the incident electromagnetic wave, the magnitude and phase of which can be represented as txy and ∅xy. These parameters txy and ∅xy are highly dependent on the geometry of the unit cell. Using the proposed design idea, dimension *S* and the size of the square patches *C*1 × *C*1 and *C*2 × *C*2 can be tuned in such a way that their resonance frequencies lie within the same band. Thus, the proposed structure can transmit co-polarized (txx, ∅xx) and cross-polarized (txy, ∅xy) components. These components’ magnitudes and phases vary over the wide range of frequencies. If their magnitudes become approximately equal—i.e., |txx |≈|txy |—and their phase responses are 90° apart—i.e., ∅d = ∅xy − ∅xx = 2nπ ± π/2—then, n being a whole number, the outgoing wave will be a CP wave. The linear polarization to circular polarization conversion performance can be represented by an axial ratio (AR), as in Equation (1):

(1) $$AR=\sqrt{\frac{{\left|t_{xx}\right|}^{2}+{\left|t_{xy}\right|}^{2}+\sqrt{a}}{{\left|t_{xx}\right|}^{2}+{\left|t_{xy}\right|}^{2}-\sqrt{a}}}.$$

Whereas a can be computed from Equation (2), as follows:

(2) $$a={\left|t_{xx}\right|}^{4}+{\left|t_{xy}\right|}^{4}+2{\left|t_{xx}\right|}^{2}{\left|t_{xy}\right|}^{2}\cos(2\phi_{d}).$$

For an ideal polarization converter, transmitted orthogonal components are exactly equal—i.e., ∅d = ∅xx − ∅xy =2nπ ± π/2—and their phase angles are exactly 90° apart. In this case, the outgoing transmitted wave is a perfectly circularly polarized wave, resulting in AR being 1 (0 dB). However, in practical systems, an AR value of 3 dB is acceptable for most systems.

**Figure 2:**
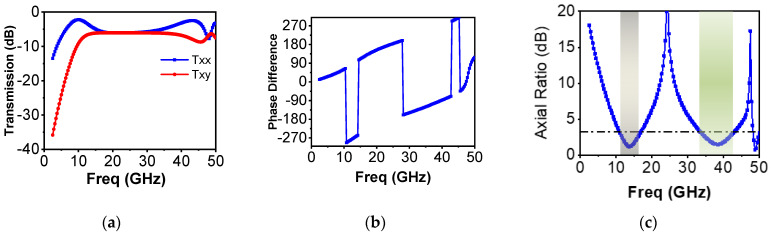

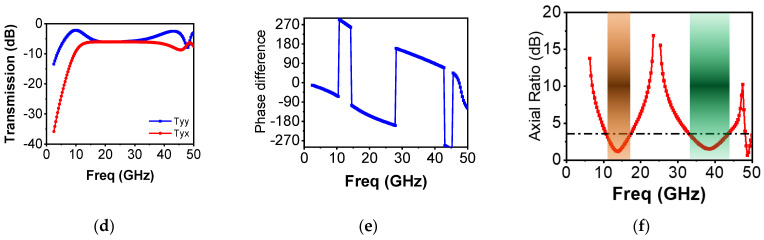
Simulated transmission response for the proposed metasurface with incident horizontal (x)polarized wave: (**a**) transmission component; (**b**) phase difference; (**c**) axial ratio. Simulated transmission response with incident vertical (y)polarized wave: (**d**) transmission components; (**e**) phase difference; (**f**) axial ratio (AR).

Figure 2a,b show that |txx| ≈ |txy| from 11.05 to 16.75 GHz and 34.16 to 43.03 GHz, and the phases of the two transmitted orthogonal components are approximately −90° or +270° (within ±15° variation). Therefore, the condition required for linear to circular polarization conversion is fulfilled exactly at some frequencies [24], whereas, for a range of frequencies, the transmitted components are not exactly but approximately equal (in this case, the transmitted wave is not perfectly circularly polarized but slightly elliptically polarized). Figure 2c presents the polarizer’s performance in terms of axial ratio. Since the axial ratio is lower than 3 dB within 11.05~16.75 GHz and 34.16~43.03 GHz, the transmitted wave’s AR condition for linear to circular polarization conversion is maintained. Moreover, from 11.05 to 16.75 GHz, the cross-polarized component (Tyx) is ahead of the co-polarized component (Txx) (phase difference > 0), resulting in a left-handed circularly polarized (LHCP) transmitted wave. Further, from the frequency range 34.16~43.03 GHz, the cross-polarized component lags behind the co-polarized component (phase difference <0); consequently, a right-handed circularly polarized (RHCP) wave is transmitted. Operational bands are highlighted as gray and dark green zones in Figure 2c. Interestingly, an additional advantage of the proposed structure is that it maintains its conversion performance when an incident vertical (y-polarized) wave is applied as an incident wave. However, this time, the resulting outgoing polarization sense is reversed; i.e., there is an RHCP and LHCP for the 11.05~16.75 GHz range (Ku band) and 34.16~43.03 GHz range (Ka-band), respectively (as shown in Figure 2d–f).

**Figure 3:**
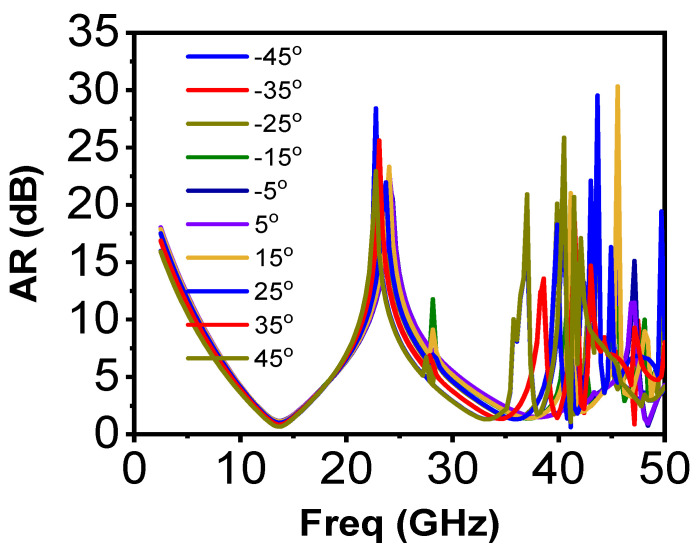
Simulated response for oblique incidences.

Since the structure of the proposed polarizer is uni-layered, it can be insensitive to large changes in incident angles. Usually, while operating in transmission modes, such converters need to bear these changes and sustain performance for oblique incident angles. To investigate this, the unit cell was analyzed in Ansys HFSS using the change in incident angles of the applied TE wave, and the response variation in terms of the axial ratio was also analyzed. The results are presented in Figure 3 in steps of 10° from 45° to −45°. It is clear that the polarizer performed equally well in the first band of operation (Ku band), which is a remarkable achievement for a transmission-based polarizer. For the second frequency band of operation, the performance in the lower half of the operational band remained unchanged, except for a small frequency shift. However, the polarization conversion performance degraded greatly for ±45° in the operational frequency band higher than the center frequency of band. This performance remained good for ±30° changes in incident angles. Thus, the proposed conversion performance remained stable for ±45° for the first band of operation and ±30° for the second band.

**Figure 4:**
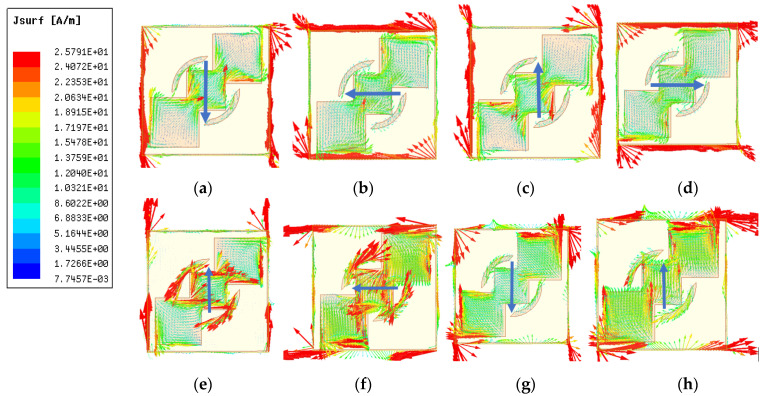
Surface current phenomenon in the proposed structure at 13.58 GHz: (**a**) t = 0, (**b**) t = T/4, (**c**) t = T/2, (**d**) t = 3T/4; and at 40 GHz: (**e**) t = 0, (**f**) t = T/4, (**g**) t = T/2, (**h**) t = 3T/4.

To explain the physics behind linear to circular polarization conversion, surface current vectors were monitored at different time intervals: t = 0, t = T/4, t = T/2, t = 3T/4, with T being the time period for the incident wave at resonant frequency. Figure 4a–d show surface current vectors at 13.58 GHz, whereas Figure 4e–h show surface current vectors at 40 GHz. Figure 4a shows that, at t = 0, the resultant surface current vectors at the unit cells were directed at an angle of 270° from the +x-axis. For ease of understanding, we represent the magnitude of these vectors at the center with the help of a blue line, with an arrowhead showing the direction of surface currents. Figure 4b shows that, at t = T/4, the surface current vectors lay along the -x-axis, manifesting a rotation of 90° from t = 0. At t = T/2, the angle changed to 90° along the x-axis. For t = 3T/4, the surface current vectors were aligned with the x-axis, hence forming an angle of 0° along the x-axis. Thus, increasing the time interval by T/4 results in further rotations of the surface current vectors by an angle 90°.

This rotation of the current vectors was clockwise, meaning the outgoing wave was an RHCP wave; it is also well-explained electrically in Section 2, Figure 2b. Similarly, surface current vectors for other operational band at the center frequency of 40 GHz are represented in Figure 4e–h at different time intervals. In these figures, it is clear that, with every time duration of T/4, the rotation of surface currents is 90° counter-clockwise, resulting in an LHCP transmitted wave, as explained electrically in Figure 2e.

## 4. Discussion

**Figure 5:**
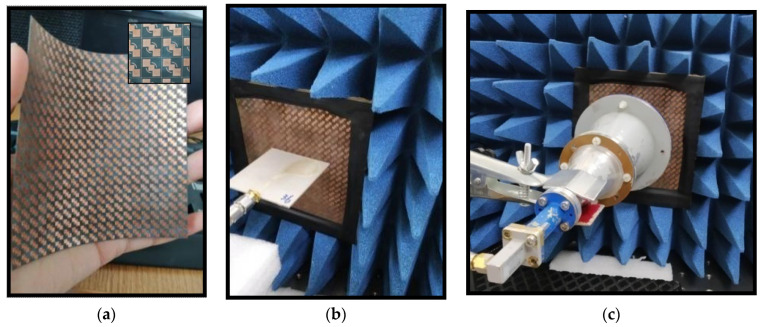
(**a**) Fabricated polarizer. (**b**) Ku band and (**c**) Ka band test setup for the polarizer.

To verify the performance of the proposed polarizer, fabrication was carried out on a 0.127 mm thick, flexible Rogers-5880 substrate using LPKF E33. Before carrying out fabrication, we made sure that the machine’s precision would not deteriorate the response of the converter by carrying out a sensitivity analysis. An array consisting of 30 × 30 unit cells was fabricated. A picture of the fabricated sample is presented in Figure 5a. To perform measurements for the polarizer, a pair of ultra-band transmit and receive antenna covering both operational bands were needed. Due to the unavailability of such antennas, measurements were completed in two sets using two different antennas. For Ku band measurements, two broadband patch antennas covering the required band of operation (11.05~16.75 GHz) were used. For measurements in the Ka band, a set of standard circular horn antennas operating in the 26.5~40 GHz frequency band were used.

**Figure 6:**
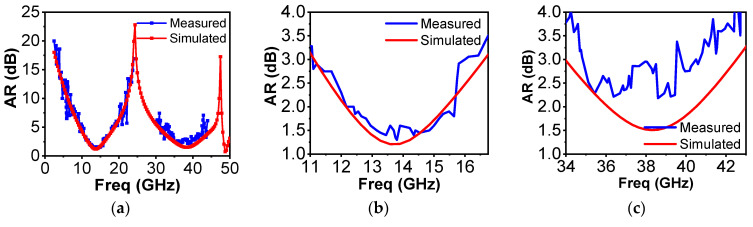
(**a**) Measured axial ratio response; (**b**) AR within Ku operational band; (**c**) AR within Ka operational band.

For the measurements of the transmission parameters of the proposed structure, an AV3672C vector network analyzer (VNA) was used. The measurement setup for the proposed converter was as shown in Figure 5b,c for the Ku and Ka bands, respectively. For measurements, a free-space characterization technique was used. After calibrating the network analyzer, the first step in free-space characterization, the reference measurements were taken. Then, as a next step, the fabricated polarizer was placed in between the transmit and receive antennas. It was ensured that the placement of the polarizer was in the line of sight of the antennas. Subsequently, measurements for linear transmission components |txx| and |txy| and their respective phases were carried out. To determine the cross-linearly polarized components (txy), the receive antenna was rotated 90 degrees. As the last step, |txx| and |txy| were calculated by determining the difference between the linear transmission components and reference measurements. Furthermore, their phases were calculated by subtracting them from reference phases. In this way, losses due to surroundings and measuring cables were compensated. Finally, the measured results were as presented in Figure 6a–c.

The measured performance in the first band of operation (Ku band) agreed well with the simulated axial ratio results, with AR values in the range from 1.5 dB to 3.5 dB and simulated values from 1.2 dB to 3.0 dB. Although the measured values of the AR in the second band of operation (Ka band) were higher than the simulated values, the trend was nevertheless the same. In the Ka band, the values of AR were in the range from 2.0 to 4.0 dB and the simulated values in the range from 1.5 to 3.0 dB. It was assumed that the frequency shift in the Ka band of operations might have been due to the fabrication tolerance of LPKF E33. Higher frequency implies more sensitivity towards minute changes in dimensions; meaning that the Ka band was more sensitive than the Ku band. This slight shift in operating frequency band may have been the result of small changes in the dimensions during fabrication of the prototype polarizer. Moreover, the non-ideal conditions, such as the finite array size of the fabricated structure as compared to the infinite array in simulations, and non-ideal experimental conditions are the reasons behind the differences in the simulated and measured results.

The comparison of the proposed structure with other already presented dual-band MS-based polarizers is presented in Table 2. The structure performed over the widest dual bands of operation despite being ultra-thin, and it showed stable performance over a wide range of oblique incident angles.

## 5. Conclusions

In summary, a broadband polarizer was here presented using single-layered transmissive metasurfaces that can perform conversion operations in dual bands (11.05~16.75 GHz, 34.16~43.03 GHz: Ku/Ka band). The structure can perform transmission polarization conversion over a broad range of frequencies (bandwidths of 41% and 23%) for dual bands. It can convert linearly polarized waves into right-hand circularly polarized waves in the Ku band and left-hand circularly polarized waves in the Ka band. To validate the design strategy, a sample of the proposed polarization converters consisting of an array of 30 × 30 unit cells was fabricated. Measurements were performed using free-space characterization techniques with a VNA and two sets of linearly polarized antennas. Simulated and measured results were coherent, which shows that the polarizer had remarkable performance in the polarization conversion operation. Such converters can be potentially applied for miniaturization and polarization control. Moreover, its polarization conversion performance using a single-side layered structure in dual bands may have diverse applications in wireless communication systems, including multiple-channel operations.

The proposed dual-band polarizer integrated with a separate feeding structure can be used in different scenarios, such as vehicles on the move, communication systems, and satellite communication systems. Since the design is a universal design, the polarizer can be redesigned to operate in any frequency band of operation for integration with dual-band LP array structures to produce dual-band CP waves. In the future, tunability will be introduced using external controlling mechanisms. For example, controllable circuit elements, state-changing materials, and structural changing scenarios in the polarizers could result in reconfigurable dual-band polarizers.

## Figures and Tables

**Table 1:** Design parameters for the proposed polarizer.

*w*2	*C*1	*C*2	*w*1	*P*	*S*	*d*	*h*
0.05 MM	1.9 mm	1.5 mm	0.2 mm	5.1 mm	1.8 mm	2.3 mm	0.127 mm

**Table 2:** Performance comparison with other state-of-the-art dual-band polarization converters (LC-to-CPs).

Ref	Frequency of Operations (GHz)	Operational Bandwidth (%)	No. of Layers	Operational Modes	Stability with Change in Angles
[21]	19.6, 29.6	4, 2.7	3	Same	-
[23]	19.95, 29.75	2.5, 1.7	3	Orth.	30°
[24]	7.6, 13	31.6, 13.8	4	Same	±25°
[25]	17.8, 36.5	25, 16.4	2	Orth.	-
[31]	20.6, 29.2	12.5, 8.7	6	Orth.	±45°, ±30°
[28]	18.5, 28.5	24, 11	2	Orth.	-
This work	13.9, 38.59	41, 23	1	Orth.	±45°, ±30°

## Data Availability

The study did not report any data.
